# Electric signal synchronization as a behavioural strategy to generate social attention in small groups of mormyrid weakly electric fish and a mobile fish robot

**DOI:** 10.1007/s00422-021-00892-8

**Published:** 2021-08-16

**Authors:** Martin Worm, Tim Landgraf, Gerhard von der Emde

**Affiliations:** 1 Neuroethology/Sensory Ecology, Institute for Zoology, University of Bonn, Meckenheimer Allee 169, Bonn, Germany; 2 Present Address: Stingray Marine Solutions AS, Stålfjæra 5, 0975 Oslo, Norway; 3 Dahlem Center for Machine Learning and Robotics, Department of Mathematics and Computer Science, Freie Universität Berlin, Berlin, Germany

**Keywords:** Weakly electric fish, Animal–robot interaction, Electro-communication, Signal synchronization, Social attention

## Abstract

African weakly electric fish communicate at night by constantly emitting and perceiving brief electrical signals (electric organ discharges, EOD) at variable inter-discharge intervals (IDI). While the waveform of single EODs contains information about the sender’s identity, the variable IDI patterns convey information about its current motivational and behavioural state. Pairs of fish can synchronize their EODs to each other via echo responses, and we have previously formulated a ‘social attention hypothesis’ stating that fish use echo responses to address specific individuals and establish brief dyadic communication frameworks within a group. Here, we employed a mobile fish robot to investigate the behaviour of small groups of up to four *Mormyrus rume* and characterized the social situations during which synchronizations occurred. An EOD-emitting robot reliably evoked social following behaviour, which was strongest in smaller groups and declined with increasing group size. We did not find significant differences in motor behaviour of *M. rume* with either an interactive playback (echo response) or a random control playback by the robot. Still, the robot reliably elicited mutual synchronizations with other fish. Synchronizations mostly occurred during relatively close social interactions, usually when the fish that initiated synchronization approached either the robot or another fish from a distance. The results support our social attention hypothesis and suggest that electric signal synchronization might facilitate the exchange of social information during a wide range of social behaviours from aggressive territorial displays to shoaling and even cooperative hunting in some mormyrids.

## Introduction

Being active and social at night are challenging for animals since vision is not available for orientation and intra-specific communication. Nocturnal weakly electric fish from Africa (Mormyridae) and South America (Gymnotiformes) found a solution to this problem by evolving an active electric sense (von der Emde and Zeymer 2020; Crampton 2019), which not only allows these fish to orient and navigate in complete darkness (von der Emde and Ruhl 2016) but also to communicate with conspecifics (Arnegard et al. 2010). Mormyrids can actively produce electric signals (electric organ discharges, EOD) with an electric organ in their tail, which has evolved from former muscle cells. Mormyrids are so-called pulse fish (Kramer 1990b), i.e. they emit brief pulses of electric signals with a rather constant, species-specific and sometimes sex-specific waveform but highly variable inter-discharge intervals (IDI). The EOD waveform changes only slowly depending on age, reproductive state and sometimes dominance relationships of the sender individual (Carlson 2016; Hopkins 2009; Terleph and Moller 2003). In contrast to EOD waveforms, individuals can rapidly vary the IDI between single EODs. For example, some mormyrids can be silent for several seconds, and then suddenly start pulsing at frequencies of up to 100 Hz (Grant et al. 1999). These variable temporal patterns of IDIs convey information about the current motivational and behavioural state of the sender in different behavioural situations (foraging, resting, etc.) and during social interactions (aggression, reproduction, group interaction, etc.) (Pannhausen et al. 2018; Carlson and Hopkins 2004; Moller 1970). Since weakly electric fish use their EODs for both electro-communication and active electrolocation, it may be difficult in some cases to decide which of these two functions controls the current emission pattern of electric signals of a fish.

Mormyrids can perceive their own signals as well as those of neighbouring electric fish with two types of epidermal electroreceptor organs distributed over their skin surface. Mormyromast electroreceptor organs respond to the self-produced EODs and to distortions induced by objects in the electric field, enabling the animals to probe their immediate environment, and thus perform active electrolocation (von der Emde and Zeymer 2020; von der Emde et al. 2010). The second type of electroreceptors (*Knollenorgans*), are highly efficient time-coders and allow mormyrids to perceive even slightest differences in the EODs of other electric fish as well as the variability in a conspecifics IDI patterns (Baker et al. 2013). They thus form the basis for electro-communication. Several IDI patterns play a crucial role in mormyrid social behaviour. Among these are so-called ‘double-pulses’, ‘discharge accelerations’, short or long ‘pauses’ of EOD production or ‘regularizations’ of IDI duration, who all appear to function as signals during electro-communication (Worm et al. 2018a; Gebhardt et al. 2012a; Carlson and Hopkins 2004).

**Fig. 1:**
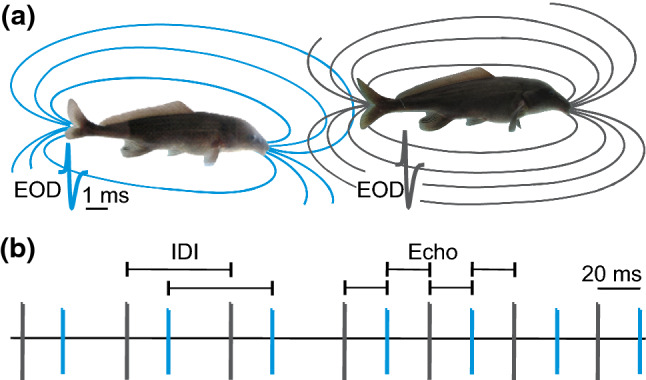
Schematic illustration of electric signal synchronization by mutual echo responses in mormyrids. **a** Each individual fish is surrounded by a dipole-like electrical field that persists for the duration of the periodically emitted electric organ discharge (EOD) outlined in black and blue, respectively. Insets represent waveform and duration of the EOD of *M. rume*. **b** Timeline of a synchronization event with vertical bars representing colour-coded EODs of both fish. The inter-discharge interval (IDI) represents the duration between signals of the same fish. The echo response describes a fixed latency with which one fish responds to the EOD of a conspecific. The echo response in *M. rume* has a latency of approximately 20 ms. An individual (blue) can synchronize its discharges to another’s (black) by echoing a successive sequence of EODs. Mutual synchronization arises when the second individual (black) starts to also respond with 20 ms echoes to the EODs of the first (blue). This results in a regularization of the IDI of each fish

**Fig. 2:**
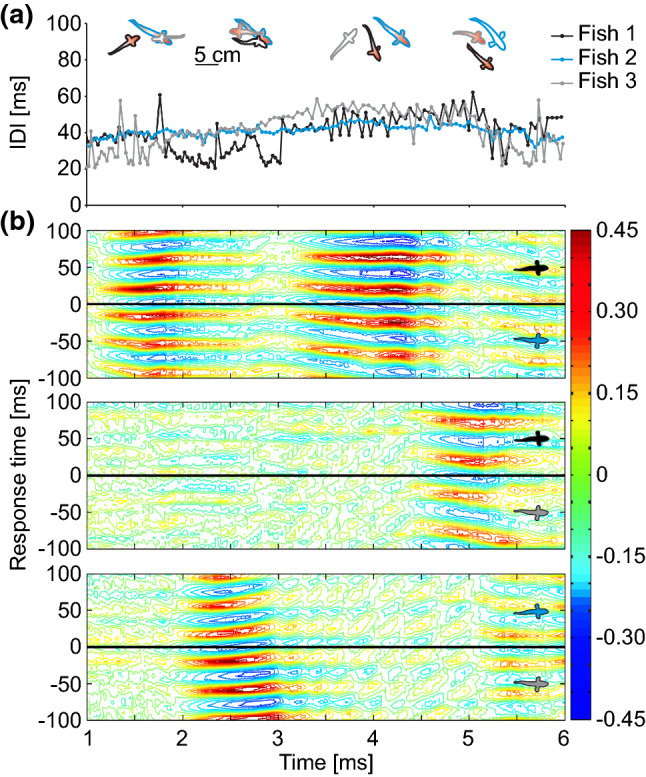
Rapid switching of synchronization between mormyrids in a group. Representation of a short interaction sequence of three *M. rume* during feeding. **a** IDI sequences of each fish, with line colours corresponding to the outlines of the inset drawings above. Insets depict snapshots from the interaction episode, where synchronizing fish are marked in red. **b** Cross-correlation analyses of all possible pairs of electric signalling interactions visualize the time course of electrical synchronization events between individuals in the group. Correlation coefficients at a particular response time are color-coded and reveal strong mutual synchronizations at the response time corresponding to the echo latency of *M. rume.* For each of the tree panels, correlation coefficients represent reactions of the color-coded individuals indicated as insets on the right-hand side of the diagrams to the respective other fish in the same panel. Data were obtained and analysed according to the procedures described in (Gebhardt et al. 2012a)

Electro-communication in mormyrids also involves interactive electrical discharge behaviours. For example, during the ‘echo response’ a fish responds to an EOD of a conspecific with the emission of a signal of its own at a fixed ‘preferred latency’ of only several milliseconds (Kramer 1974; Russel et al. 1974; Carlson 2002; Worm et al. 2017). Continuous echo responses during a social encounter result in a ‘synchronization’ of the discharges of two individuals, particularly so if both individuals echo each other’s EODs mutually (Fig. 1) (Worm et al. 2018b). In groups of several fish, individuals were found to switch synchronization partners within short time frames, resulting in dynamically changing interactive patterns (Fig. 2). Such synchronization episodes were suggested to maintain group coherence or even enable mutual recognition among members of a group (Arnegard and Carlson 2005). Echo responses may also serve as a jamming avoidance strategy during active electrolocation (Heiligenberg 1976; Schuster 2001). Based on studies with an interactive fish robot that mimicked the mormyrid echo response by means of electric playback of the EOD, we have previously suggested that mutual synchronization of EODs by echoing might constitute a communication strategy which allows a mutual allocation of social attention, and thus, the exchange of information during an encounter of two fish (Worm et al. 2018b). In the current study, we chose the same experimental approach with the mormyrid *Mormyrus rume proboscirostris* to show that the proposed communication framework could be a strategy to open a brief communication window, particularly in the electrically noisy environment of a social group.

Research on how animals communicate, form coherent groups and coordinate their actions has greatly benefited from cooperations between behavioural biology and robotics. Robots allow biologists to systematically develop, test, and refine hypotheses about animal behaviour because the robot, unlike live animals, is completely under the experimenter's control and allows for exact reproducibility of trials and precise standardization of experimental conditions (Webb 2002; Krause et al. 2011; Romano et al. 2019).

This is particularly useful in behavioural experiments where robots are used to trigger responses from live animals. Playbacks of behavioural displays can be presented, for example as acoustic or visual stimuli, and allow to restrict stimulus presentation to a defined sensory channel (McGregor 2000). Mormyrids are especially amenable to electrical playbacks of their EOD because of the importance of this signal for social communication (Kramer 1990a).

To test which features a robotic fish must have to be accepted as a conspecific in a group of *M. rume*, we have presented a biomimetic weakly electric fish robot to live fish (Donati et al. 2016). By combining motility cues with electric signalling displays, we could show that the robot was able to recruit both single individuals and groups of *M. rume* from a shelter into an exposed area. However, while visual and motility cues were not necessary to induce this behaviour, EOD emissions were. In further experiments that eventually reduced the robot to its electric playback signal from the fish’s perspective (Worm et al. 2017, 2018a), we showed that mormyrids can rely on only electro-perception to perceive and localize both the locomotor behaviour and the electro-communication signals of conspecifics. Visual cues or short-distance cues sensed through the lateral line were not found to be necessary, since a moving, but invisible and body-less electrical signal source was also sufficient to evoke the full spectrum of social interactions in the fish (Worm et al. 2018a). Electrical playback of the mormyrid EOD is, therefore, a powerful tool to socially integrate robotic dummy fish into groups of live mormyrids (Pannhausen et al. 2018).

Most biomimetic robots produce social cues detached from the behaviour of interaction partners, an approach commonly referred to as ‘open-loop’ (Romano et al. 2019). However, the acceptance of artificial group members, and eventually the study of more complex social interactions, may require closing the feedback loop, allowing the robot to respond in real-time to actions of live conspecifics (Landgraf et al. 2021). In a previous work, we closed the feedback loop between fish and robot at the level of electrical communication in experiments with a live electric fish and a robot that could be freely moved on user-defined trajectories (Worm et al. 2018b). In this study, the robot was able to produce echo responses to the fish’s EOD and could thereby synchronize electrically with the fish. This strongly enhanced back-synchronizations of EOD-sequences by *M. rume* with the mobile robot in both frequency and duration when compared to a static control playback. This frequently occurred when the test fish approached the robot.

We therefore argued that interactive signalling through echoing of a conspecific’s EODs may constitute a behavioural mechanism by which mormyrids can specifically allocate social attention between two nearby individuals during electro-communication and constitute a foundation for complex social behaviours (Worm et al. 2018b). However, since these experiments involved only single live fish, it remained unclear whether echo responses between the robot and a focal individual have an effect on other animals in the group.

Here, we consequently extend our findings to the context of group interactions and measure the influence of a mobile dummy on the motor and electromotor behaviour of small groups of two, three, and four *M. rume*. To specifically identify behavioural situations in which mormyrids synchronize their electrical discharge activity, we take advantage of simultaneous recordings of video and electric discharge activity and provide quantitative and qualitative descriptions of the behaviour accompanying episodes of discharge synchronization. Our results are consistent with previous experiments on pairwise interactions and in addition highlight the importance of closing the feedback loop on the motor level as well. These findings also support the hypothesis that mormyrids can use the echo response to address a specific individual within a group and establish a periodic communication framework based on mutual discharge synchronizations. Further experiments should therefore be designed with the presumptive nature of exchanged signalling information in mind.

## Methods

### Animals

The experiments presented in this paper constitute an extension of the research design described in Worm et al. (2018b). In total, 23 captive bred *Mormyrus rume proboscirostris* were used in these experiments. Fish measured between 6.4 and 11.4 cm in standard length and were kept in 50–200 L holding tanks at a temperature around 25 °C, a water conductivity of approximately 100 µS cm^−1^, and a light/dark cycle of 12/12 h. Individual fish were confined to separate compartments within the holding tanks. Each compartment provided a shelter and was connected to at least one neighbouring compartment by a water permeable barrier that prevented physical contact but enabled electro-communication between individuals. Animals were fed at least five times per week with defrosted chironomid larvae.

Behavioural experiments were performed with single fish and small groups of two, three, and four individuals. Nine groups (*n* = 9) were tested for each group size. Since the number of research animals was limited, it was necessary to test individual fish more than once for several group sizes. Groups were composed of similarly sized animals, and experimental trials were arranged to assure that no animal was tested more than once per day. Fish were individually marked at their caudal and/or pectoral fins to make sure that groups were composed of the same animals across experimental sessions. The tested fish were juvenile at the time of experimentation and their sex could not be reliably determined.

All experiments were carried out in accordance with the guidelines of German law, with the animal welfare regulations of the University of Bonn, and with the ‘Guidelines for the treatment of animals in behavioural research and teaching’ (ASAB 2018).

### Experimental setup

**Fig. 3:**
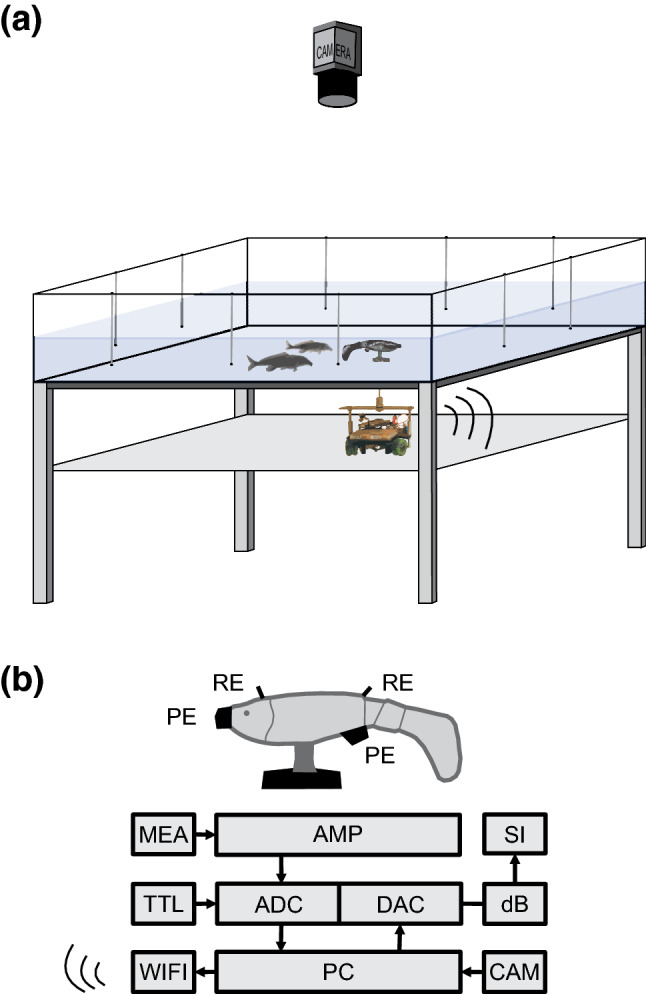
Experimental setup. **a** Fish tank with multi-electrode array and video camera for recording electrical signals and swimming behaviour during experiments. The mobile dummy fish is coupled via a magnet to the WIFI-controlled wheeled robot on the plane underneath the tank. **b** Schematic of the dummy fish and technical components. MEA: Multi-electrode array consisting of five pairs of recording electrodes in the tank. AMP: differential amplifier. ADC: analogue to digital converter. TTL: Trigger box receiving input from the recording electrodes (RE) on the dummy fish inside the tank. A TTL pulse is generated for each EOD registered at these electrodes to trigger a playback EOD at the latency corresponding to the echo response. PC: computer for data acquisition, playback generation and control of the robot’s trajectories within the tank. CAM: video camera for behavioural recordings. WIFI: wireless communication with the robot underneath the tank. DAC: digital to analogue converter. DB: signal attenuator. SI: analogue stimulus isolation unit powering the electrical signal that is generated at the playback electrodes (PE) of the dummy fish

All experiments were performed in a 120 cm × 100 cm × 20 cm tank. The tank was mounted on top of a metallic support frame in a way that left the base area accessible from underneath (Fig. 3). To shield the animals from outside disturbances and provide the best possible contrast for video analysis, the tank walls were covered from all sides with white cardboard and the base plate was covered with white self-adhesive foil on the inside. The water was heated to 26 ± 1 °C and water conductivity was adjusted to 100 ± 5 µS cm^−1^ for all experiments. All maintenance equipment was removed before every experimental session and the water level within the tank was kept at 15 cm. A second plane was installed below the tank. This plane supported a wheeled robot (Landgraf et al. 2012) that could be steered via a wireless connection to move on arbitrary trajectories below the tank.

A dummy fish, made from an 8 cm fishing bait, was fixed on a base plate that was mounted on a small magnet. This dummy was equipped with two pairs of electrodes. A pair of carbon electrodes for playback generation was inserted into the rubber at the snout and the rear end of the fishing bite and a pair of silver electrodes, for recording electric signals generated by the fish was inserted dorsally along the longitudinal axis. From the centre of the robot underneath the tank, a neodymium magnet was held up to the tank’s base plate, where it coupled to the dummy's magnet, thus enabling the control of the dummy’s trajectories within the tank via the remote controlled robot below the tank.

A multi-electrode array with five pairs of carbon electrodes was evenly distributed along the inside walls of the tank and recorded all electrical activity in the water. Electrical signals were differentially amplified (Brownlee Precision Model 440, Palo Alto, CA), digitized (CED Power 1401, Cambridge Electronic Design, Cambridge, UK), and recorded to disc using Spike2 software (Version 5.21, Cambridge Electronic Design, Cambridge, UK). Simultaneously, all behaviour within the tank was recorded at 15 fps with an infrared sensitive video camera (DMK 23FM021 FireWire Camera with Vari Focal T4Z2813CS-IR CCTV Lens, The Imaging Source, Bremen, Germany) using the Spike2 Video Recorder. The tank was illuminated indirectly with a LED floodlight and light levels were adjusted to 1.5lx of visible light intensity (Light ProbeMeter™, 403 125, Extech Instruments) at the water surface in the centre of the tank. Camera vision was enhanced by additional illumination with infrared spotlights (850 nm, IR Illuminator Model SA1-60-C-IR, Itakka, Wattens, Austria).

The silver electrodes of the dummy were used to record signals of live fish that came into close range of the dummy. These signals were amplified differentially using a custom build trigger-box (University of Regensburg), which generated a TTL pulse for each signal exceeding a threshold determined by amplification. This TTL-output was used to generate interactive electrical playback mimicking the mormyrid echo response in real-time via the CED 1401 and the Spike2 sequencer.

To generate electrical playback signals of the EOD inside the water, the dummy's carbon electrodes were connected to a stimulus-isolation unit (model 2200, A-M Systems Inc., Carlsborg, WA) as a power supply. Playback EODs were output via the Spike2 sequencer, converted from digital to analogue using the CED 1401, and adjusted with a dB-attenuator (University of Regensburg) to match the EOD-amplitude of a medium sized fish.

Behavioural trials were performed with two types of electrical playback sequences, a static random playback with predetermined IDI sequences and a dynamic echo playback that would mimic the mormyrid echo response based on behavioural input from the fish during experiments. These playbacks were assembled from a pre-recorded template EOD that was obtained by averaging 50 EODs of a *M. rume*. Template EODs were recorded head-to-tail using a 1 Hz high-pass filter and digitized at a sampling rate of 50 kHz. Static random playback sequences were generated by assembling template EODs to sequences of 15 s duration. IDIs for these sequences were randomly selected within two standard deviations around the mean (67 ms) of a distribution containing 17,644 IDIs that were previously recorded from live fish. Random playbacks were repeated three times to obtain a 45-s stimulus protocol and a new sequence was designed for every trial to avoid pseudoreplication. Dynamic echo playbacks were designed to respond to electrical signals of live fish, thus enabling mutual discharge synchronisation between dummy and *M. rume*. Echo playbacks were generated by programming the Spike2 sequencer to output playback EODs at intervals greater than 60 ms in the absence of a trigger signal and respond by generating a playback EOD with a latency of 21 ms each time a fish's EOD was detected by the dummy’s trigger electrodes. A virtual refractory period prevented the program from echoing its own signals.

Behavioural experiments were initiated by placing single test fish or groups inside a 22 cm × 14 cm opaque start box inside the experimental tank. To habituate the fish to possible disturbances associated with the movements of the dummy and the wheeled robot below the tank, the dummy was then moved by the robot for 3 min while the fish remained inside the box. Test fish were then released from the start box to interact with the mobile dummy in three consecutive trials. These trials featured either a static playback with random intervals, a dynamic playback imitating the mormyrid echo response, or no playback as a control. The order in which these experimental conditions were presented to the fish within a session was pseudo-randomized. During the trials, the dummy was moved to first approach the fish and then turn to make them follow into the centre of the tank. Each presentation started with a 10 s period without electrical playback, followed by three 15 s episodes repeating the respective playback condition. This protocol resulted in a total of 55 s of recorded data. All relevant time points were marked with a 100 ms infrared light signal that was controlled by the Spike 2 sequencer and recorded to video to assure synchrony between video and waveform data. Additionally, trials were performed in which fish behaviour was recorded in the absence of the robot. In these trials, the dummy was removed from the tank after the habituation period and the behaviour of the test fish was then recorded according to the same time points defined for playback presentation. This additional control was performed with all group sizes in a separate experimental session on a non-consecutive day. Half of the groups were first subjected to this control in the first session, while the other half were confronted with the moving dummy in their first session.

### Data analysis

All videos were rectified using open CV camera calibration software prior to the analysis to compensate for radial distortions. Video images were then used to obtain Cartesian coordinates for all fish and the dummy in three-second intervals. This yielded 15 measurements per fish and trial for each experimental condition. At the same time points, the shortest distance between the snout of each fish and the closest wall of the tank was determined. These measurements were performed in ImageJ (version 1.46r, National Institutes of Health, USA). Because it was not possible to identify individual fish consistently across the successive trials of an experimental session solely based on video recordings, values obtained from individual fish were not differentiated. Instead, the analysis was based either on mean values or minimum/maximum distances. Nearest neighbour distances (NND) between dummy and fish, as well as between the fish, were calculated from Cartesian coordinates using Matlab (Version R2013b, The MathWorks Inc. Natick, MA).

The number of the robot's turns that were followed by at least one fish in response to the different experimental conditions were quantified manually from video recordings. Observer bias was excluded by randomizing file names during the analysis, leaving the experimenter blind to the playback condition during which the video had been recorded.

Statistical comparisons were performed using SPSS (version 22.0, IBM Corp., Armonk, NY). Repeated-measures designs were used for comparisons within the different group sizes. A repeated-measures ANOVA was performed if data were assumed to be normally distributed based on Shapiro–Wilk's test. A non-parametric Friedman test was used on data that did not meet this assumption.

To identify specific behavioural situations in which mormyrids synchronize their electrical discharge activity, all experiments with a group size of two that involved electrical playback were screened for episodes during which both test fish showed episodes of following the mobile dummy. Waveform data recorded during these episodes were converted to time series marking each EOD that was generated by either fish or playback during these sequences. EODs of the test fish were assigned to the respective sender according to the procedure described in Gebhardt et al. (2012a). This assignment to a particular sender was accomplished by manually associating amplitudes and polarities of EODs, which were recorded on multiple channels via the multi-electrode array, with the spatial positions the fish occupied in relation to the recording electrodes on a corresponding video frame. Adaptive cross-correlations for a response time of ± 100 ms were calculated over the time course of each episode for the IDI-sequences of the playback and each fish, as well as for the two fish. These analyses were performed in Matlab according to (Gebhardt et al. 2012a). The maximum correlation coefficient of electrical signalling sequences within the analysed response time frame was extracted for all possible pairings. These sequences of maximum correlation coefficients were screened for episodes with relatively high values defined by a correlation coefficient ≥ 0.3 that lasted for at least 500 ms. Behavioural patterns displayed during such episodes were further characterized through manual inspection of the corresponding video recordings. In particular, spatial relationships between synchronizing partners in the video frame corresponding to the time when the 0.3 threshold was crossed were analysed. This was done in ImageJ by determining the angle between the line connecting the centres of the communication partners and the orientation of the individual that initiated synchronization, or was synchronized to, respectively.

## Results

**Fig. 4:**
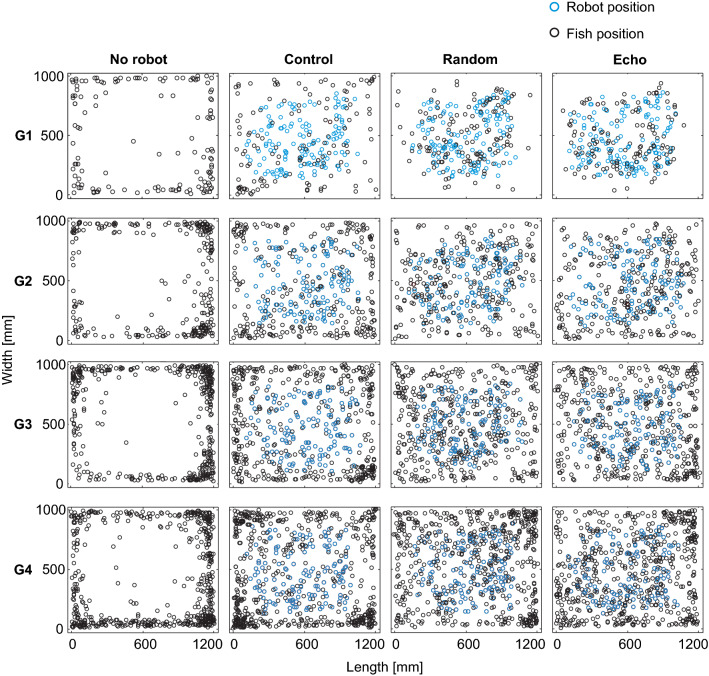
Influence of the mobile robot on fish positions in differently sized groups. Positions of fish (black) and robot (blue) inside the tank are indicated as snapshots with three-second intervals for the different group sizes (G1-G4) and experimental conditions and summed over all trials. In the absence of the robot, fish reacted to the novel environment with a strong preference for staying close to the tank walls. This behaviour persisted independently of group size. Avoidance of the central area was less pronounced in the presence of the electrically silent robot and was almost entirely abandoned by single fish during trials when the robot fish generated the static random playback or a dynamic echo playback. However, with increasing group size, the robot’s influence on fish behaviour diminished and animals increasingly resumed their preference for the area close to the tank walls

Through the confrontation of individual animals and small groups of fish with our mobile dummy fish, we could reliably induce interactive social behaviour in our experiments with *M. rume*. As expected, the mobilizing influence on real fish was strongest when the dummy emitted electrical playback EODs. The biggest effect was observed among single fish, who were most likely to abandon their preference for wall-following behaviour and interact with the dummy, whereas the influence of the robot on live fish decreased with increasing group size, even during the presentation of electrical playback. These effects of experimental condition and group size on fish positions during our experiments are illustrated in Fig. 4.

**Fig. 5:**
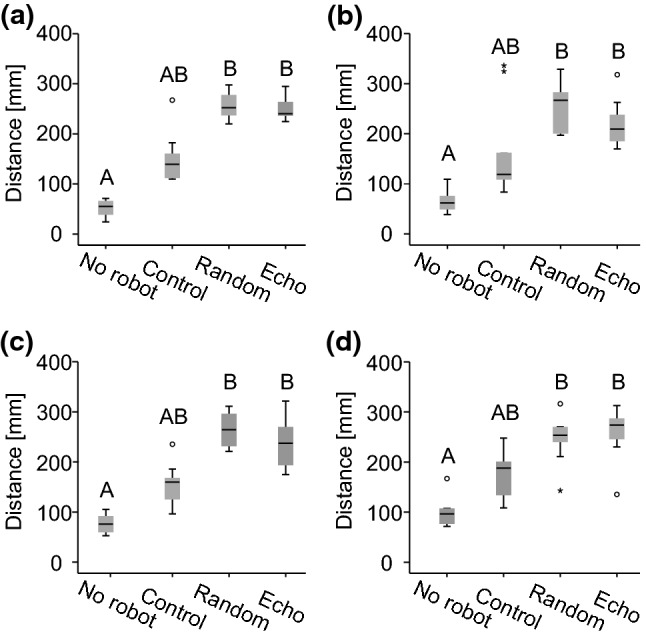
Influence of the mobile robot on wall-following behaviour in differently sized. Groups. Distance to the tank wall depending on experimental condition for trials with single fish (**a**) and the respective maximum distance, i.e. the distance of the fish that moved furthest towards the centre, for groups of two (**b**), three (**c**), and four (**c**) fish. For each group size, results from experimental conditions that do not share a common superscript letter differ significantly based on Bonferroni corrected *p*-values

As becomes apparent from the results of trials without the robot in the left-hand column of Fig. 4, all fish stayed close to the tank walls (median distances: 55–96 mm) and avoided swimming into the open area after being transferred into the unfamiliar testing environment. We therefore assessed the influence of the mobile dummy on live fish by quantifying their willingness to give up wall-following behaviour and move into the open area during experimental trials (Fig. 5). The distance between fish and the tank wall was significantly influenced by the robot in groups of all sizes (single fish: *χ*^*2*^_(3)_ = 23.13; *p* < 0.001; Fig. 5a; groups of two: *χ*^*2*^_(3)_ = 17.03; *p* < 0.001; Fig. 5b; groups of three: *χ*^*2*^_(3)_ = 21.13; *p* < 0.001; Fig. 5c; groups of four: *χ*^*2*^_(3)_ = 18.47; *p* < 0.001; Fig. 5d). In the presence of the dummy, animals were significantly more likely to move into the open area when we presented either a static random playback or a dynamic echo playback (median distances: 209–274 mm). During control trials with the electrically silent dummy, recorded distances to the tank wall (median values: 119–188 mm) were always intermediate to the control without dummy and the two playback conditions.

**Fig. 6:**
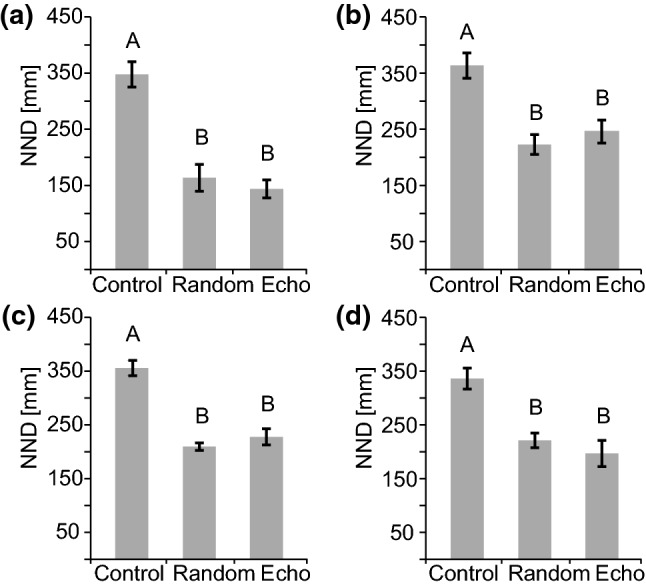
Influence of electrical playback on nearest neighbour distances. Average distance between the mobile robot and the test fish (**a**), and the robot’s nearest neighbour distance (NND), respectively, for groups of two (**b**), three (**c**), and four (**d**) fish. Comparisons were made within each group size between the electrically silent control, the static random playback, and the dynamic echo playback. Conditions with different superscript letters differ significantly based on Bonferroni corrected *p*-values

To assess the attractiveness of the robot for live fish during the different experimental conditions, we calculated nearest neighbour distances (NND) from the dummy’s perspective (Fig. 6). The experimental condition had a significant influence on the dummy's NND in all group sizes (single fish: *F*_(2, 16)_ = 51.62; *p* < 0.001; Fig. 6a; groups of two: *F*_(2, 16)_ = 16.17; *p* < 0.001; Fig. 6b; groups of three: *F*_(2, 16)_ = 33.55; *p* < 0.001; Fig. 6c; groups of four: *F*_(2, 16)_ = 19.03; *p* < 0.001; Fig. 6d). In all cases, distances were significantly longer during the electrically silent control conditions compared to the two playback conditions. The difference in effect between the two playback conditions and the control treatment was most pronounced during the trials with single fish and diminished slightly in larger groups.

**Fig. 7:**
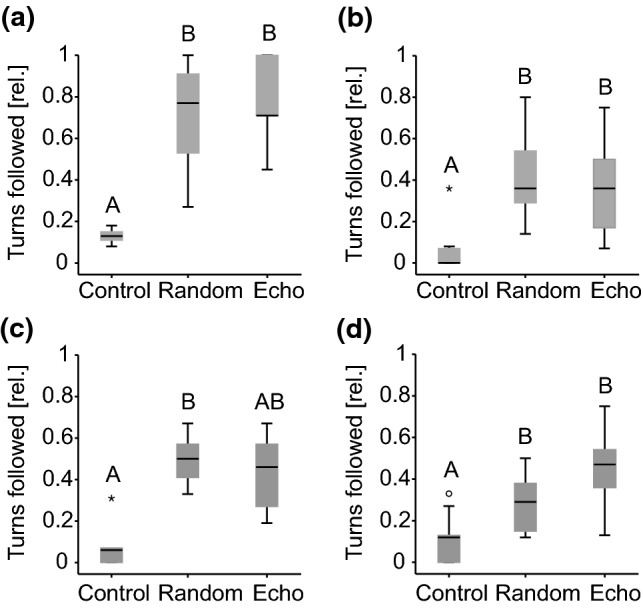
Following behaviour in responses to electrical playback. Relative number of the robot’s turns that were followed by at least one fish during the different playback conditions in tests with single fish (**a**) and groups of two (**b**), three (**c**), and four (**d**) fish. For each group size, results from experimental categories that do not share a common superscript letter differ significantly based on Bonferroni corrected *p*-values

Finally, we obtained a measure to determine whether the fish specifically followed the dummy by quantifying the relative amount of the robot's turns that were followed by at least one fish of a group (Fig. 7). In general, we observed a significant increase in the relative amount of followed turns in response to electrical playback presentation (single individuals: *χ*^2^_(2)_ = 14.11; *p* = 0.001; Fig. 7a; groups of two: *χ*^2^_(2)_ = 13.56; *p* = 0.001; Fig. 7b; groups of three: *χ*^2^_(2)_ = 12.400; *p* = 0.002; Fig. 7c; groups of four: *χ*^2^_(2)_ = 9.77; *p* = 0.008; Fig. 7d). The electrically silent control condition induced relatively little following behaviour in fish (median values: 0–0.13), which was, except for group size three, always significantly different from the two playback conditions. The dummy always elicited more turns in the following fish when it emitted either the static random playback (median values: 0.29–0.77) or the dynamic echo playback (median values: 0.36–0.71). As was observed for previous measures, single fish followed most turns in response to playback, but the willingness of any fish to show motor responses towards the dummy declined with increasing group size. On a motor level, the two playback types never elicited statistically different responses in *M. rume*.

To illuminate the behavioural context during which electric discharge synchronizations occur, we investigated mixed groups of two live fish and the robotic dummy fish in greater detail. We specifically singled out video sequences during which both fish interacted with the dummy. The number and duration of these episodes are listed for each playback condition in Table 1. In total, 112.4 s were analysed for the static random playback and 127 s for the dynamic echo playback. Discharge synchronizations during these episodes were quantified based on cross-correlation analysis between all possible pairs of recorded IDI-sequences. We identified instances of strong synchronization based on a threshold criterion defined by a correlation coefficient greater than 0.3 for a duration longer than 500 ms. During the experiments with the static random playback, this criterion was never met by randomly occurring correlations of the dummy's signals with the discharge sequences of either of the two fish. However, both fish synchronized their discharges, both to the playback and to the respective other fish.

**Table 1:** Number of analysed sequences with relatively strong synchronization in response to the static random playback and the dynamic echo playback

Playback	Dummy versus Fish 1	Fish 1 versus Dummy	Dummy versus Fish 2	Fish 2 versus Dummy	Fish 1 versus Fish 2	Fish 2 versus Fish 1	Duration [s]
Random	0	20	0	8	10	22	112.4
Echo	16	19	5	16	15	17	127

The table summarizes the number of sequences during which synchronization in *n* = 9 mixed groups of two fish and the dummy exceeded a correlation coefficient of 0.3 for at least 500 ms. Sequences are specified for the six possible pairings between the dummy, fish 1, and fish 2 of the respective group. The column on the right-hand side gives the total amount of time in seconds that was analysed for each playback condition. It represents episodes of following behaviour during the experiment with that group

Based on the defined threshold criteria for strong synchronizations, we analysed the behavioural patterns that had simultaneously been displayed by the two fish or the dummy during that part of an episode involving synchronization.

Our analysis shows that the synchronization of electric signalling sequences through echoing of another individual's EODs mostly occurred during relatively close social interactions, usually when following or approaching that individual. We observed a total of 127 behavioural episodes during which discharge synchronizations by *M. rume* to either the playback signals or the EODs of the respective other fish in the group exceeded the threshold criteria. Of the 60 episodes observed during experiments with the static random playback, four were discarded because the response time, at which synchronization occurred, did not correspond to the 20 ms latency at which echo responses typically occur in *M. rume* and because the animals did not show any interactions at the time. Of the remaining episodes, 73% were associated with behavioural situations where the synchronizing individual approached either the dummy or the other fish from behind. In about 41% of all episodes, focal fish were swimming in parallel to the trajectory of the robot or the fish that was being synchronized to. Similar behaviour occurred during episodes with the dynamic echo playback. Here, we associated 61% of all episodes with approaches and 44% with parallel swimming behaviour.

**Fig. 8:**
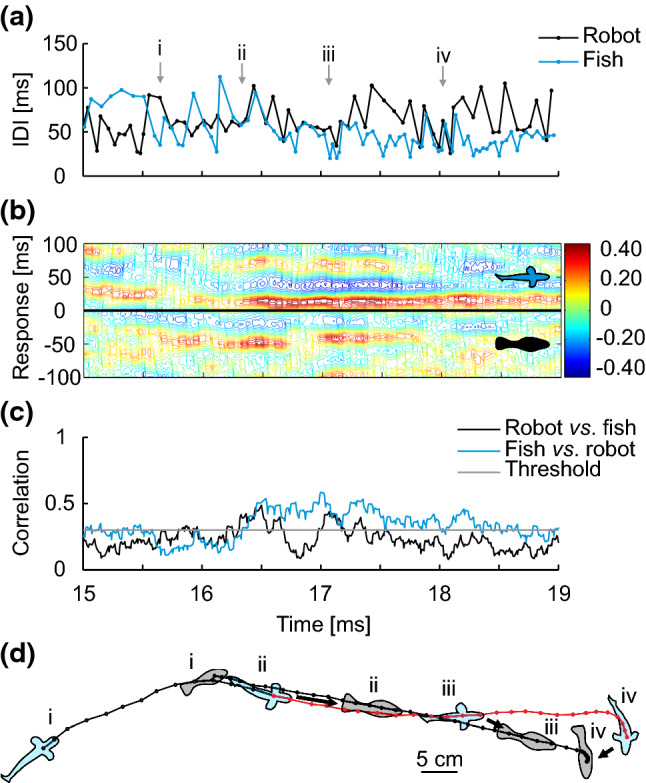
Electrical discharge synchronization to a static random playback. **a** IDI-sequences of the fish (blue) and a static random playback (black) emitted by the robot during a short interaction period. **b** Cross-correlation diagram of the sequences depicted in (**a**) with color-coded correlation coefficients for a response time of ± 100 ms. High correlations at positive response times represent discharge synchronizations of the fish (blue inset) with the dummy (black inset) at that response time. High correlations at negative response times can only occur randomly because the random playback does not respond to the fish. **c** Maximum correlation coefficients within the 100 ms response-time window in (**b**) plotted over the same time frame for correlations of the signal sequences of the fish with the robot (blue) and vice versa (black). The horizontal grey line delineates the 0.3 threshold indicative of relatively high correlation. **d** Illustrations of the interaction of fish (blue) and robot (grey) drawn to scale at several time points along their swimming trajectories. At the beginning of the episode, the fish were located at a distance from the robot (i), approached quickly, and started synchronizing its discharges to the playback when it was less than one body length away (ii). This one-sided synchronization persisted for the remainder of the whole episode, during which the fish first followed directly behind the robot (iii) and eventually caught up into a lateral position next to the robot, which then slowed down and turned (iv). Trajectories marked in red indicate that the fish was synchronizing its discharges to those of the playback with a correlation coefficient of 0.3 or higher as a part of a coherent synchronization sequence of at least 500 ms. Black arrows indicate discharge synchronization in a given situation. Behavioural descriptions from individual video frames correspond to the enumerations at fixed time points in (**a**)

**Fig. 9:**
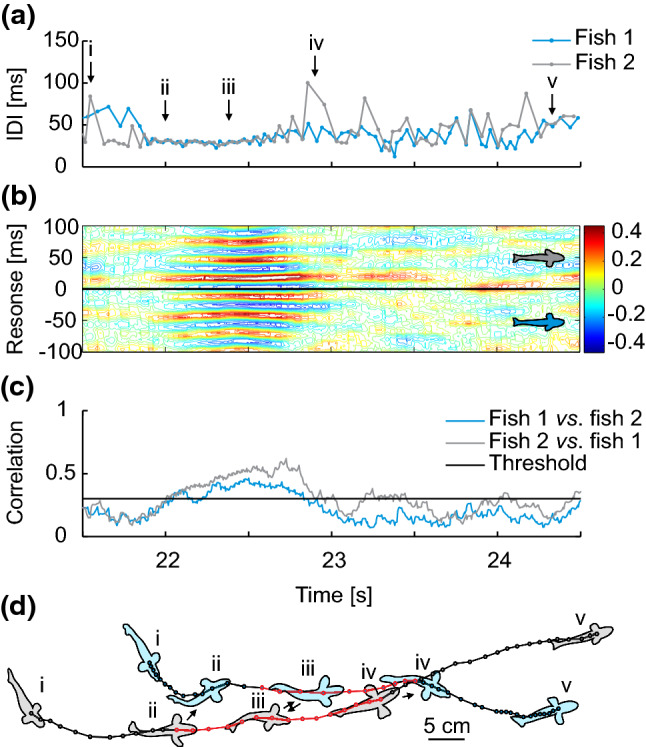
Mutual discharge synchronization between mormyrids. **a** IDI-sequences two *M. rume* during a short interaction. **b** Cross-correlation diagram of the sequences depicted in (**a**) with color-coded correlation coefficients for a response time of ± 100 ms. High correlations at positive response times represent discharge synchronizations of fish 2 (grey) with fish 1 (blue). High correlations at negative response times represent synchronization of fish 1's discharges with those of fish 2 at that response time. **c** Maximum correlation coefficients within the 100 ms response-time window in (**b**) plotted over the same time frame for correlations of the signal sequences of fish 1 with fish 2 (blue) and vice versa (grey). The horizontal black line delineates the 0.3 threshold indicative of relatively high correlation. **d** Illustration of the interactions of fish 1 (blue) and 2 (grey) drawn to scale at several time points of the interactions along their swimming trajectories. Ahead of the synchronization episode, both fish were located more than a body length apart (i). Fish 2 then approached fish 1 from behind to a lateral, almost parallel position and initiated electrical discharge synchronization (ii). This signalling behaviour was reciprocated shortly thereafter by fish 1 and culminated in a short sequence of mutual discharge synchronization and a regularization of IDI pattern (iii). Discharge synchronization was terminated by fish 2 (iv) shortly before the trajectories of the two fish crossed and they eventually stopped interacting (v). Trajectories marked in red indicate that the respective fish was synchronizing its discharges to those of the other individual with a correlation coefficient of 0.3 or higher as a part of a coherent synchronization sequence of at least 500 ms. Black arrows indicate which fish engaged in discharge synchronization in a given situation. Behavioural descriptions from individual video frames correspond to the enumerations at fixed time points in (**a**)

Exemplary episodes of typical sequences involving approach behaviour and parallel swimming are illustrated in greater detail in Figs. 8 and 9. Episodes of relatively strong EOD synchronization often occurred in situations during which *M. rume* approached another fish from behind, swam closer and then into a more lateral position next to its counterpart, eventually moving in parallel to the other’s swimming trajectory. This behaviour was frequently also directed towards the dummy. The respective motor and electromotor components of such behaviour are illustrated in Fig. 8.

Of particular interest were episodes involving mutual IDI-synchronization between two fish, i.e. episodes during which echo responses by one fish, the initial sender, in turn induced echo responses by the initial receiver to the sender's EODs. In total, we observed nine such episodes. One of these episodes were disregarded because synchronization did not occur at the response time characteristic for echo responses in *M. rume*, and animals did not obviously interact. The remaining eight episodes were all associated with behavioural situations during which one fish followed right after the other (one episode) or eventually caught up to a more lateral position relative to the ahead swimming individual (seven episodes). Interestingly, the approaching fish initiated the mutual EOD synchronization in all of these episodes. An exemplary episode of synchronization through mutual echo responses between two fish is illustrated in Fig. 9.

**Fig. 10:**
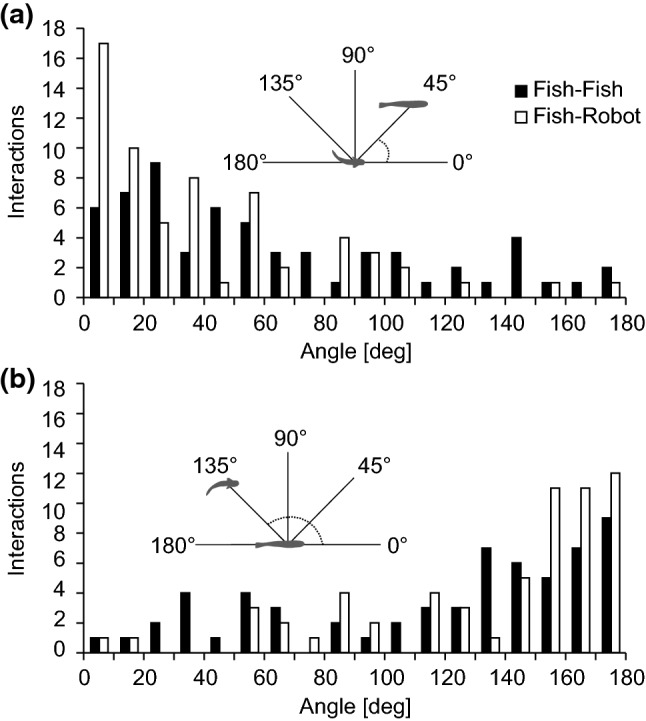
Interactions during electrical discharge synchronizations in groups. Angular relationship of two fish (black) or a fish and the dummy (white) at the onset of sequences with strong discharge synchronizations defined by a cross-correlation coefficient ≥ 0.3 and a duration of at least 500 ms. **a** Frequency of angular relationships between the swimming direction of the synchronizing fish and the connecting line to its synchronization partner. The higher frequency of low angles indicates that fish synchronized more frequently while they were faced towards their synchronization partner. **b** Frequency of angular relationships for the angle between the connecting line between the synchronizing fish and its synchronization partner, and the swimming direction of the individual whose discharges were being synchronized to by the focal fish. The higher incidence of high values indicates that individuals were being synchronized to more frequently by individuals that approached them from behind. Angular relationships of synchronization partners are illustrated for a fish–robot interaction in the insets of (**a**) and (**b**). No differentiation was made between the two playback conditions during the quantification of fish–robot interactions. Bin size: 10 degrees

We quantified these observations by determining the angular relationships between two fish or the dummy in the video frame corresponding to the point in time when a fish first passed the threshold criterion for relatively strong EOD synchronization. The frequency of angular relationships between the swimming direction of the fish that initiated a synchronization episode by emitting echo responses and the direction towards the receiver of these echoes at the time the threshold criterion was passed are shown in Fig. 10a. Both for interactions between two fish and for interactions of one fish with the dummy, the higher frequency of angles close to zero is consistent with spatial relationships where the synchronizing fish was oriented towards the individual (fish or robot) who’s EODs it echoed in that moment. The opposite is the case from perspective of the receiver of echo responses (Fig. 10b). Here, the frequencies of angular relationships between the swimming direction of the individual (or the robot) receiving echoes to its EODs and the direction towards the synchronizing fish are plotted. The higher occurrence of angles close to 180° indicates that the synchronizing fish approached the receiver from behind in the majority of cases. Spatial distances between individuals at the time the threshold criterion for synchronization was passed were relatively close (111 ± 43 mm (*mean* ± *s.e.*) during experiments with the static random playback and 114 ± 59 mm (*mean* ± *s.e.*) during experiments with the dynamic echo playback), and corresponded approximately to the presumed outer limit for active electrolocation of the test fish. Electric discharge synchronizations, thus occurred during behavioural situations when a fish approached another individual from a distance, and often resulted in social interactions between synchronization partners. Similar behaviour was observed in response to the robot fish when it emitted playback EODs. These results are consistent with the hypothesis that the synchronization of EODs, enabled by the mormyrid echo response, represents a signalling strategy to specifically single out and address another individual within a social group and communicate an intent to socially interact with that individual.

## Discussion

Our results extend the observations made in (Worm et al. 2018b) from single fish to small groups of up to four *M. rume* and demonstrate that a mobile fish robot can influence motor and electromotor behaviour of live animals based on electric playback generation (see also Pannhausen et al. 2018). Concerning the swimming behaviour of individuals in groups, the influence of an electro-communicating dummy was reliable, but the particular playback pattern did not affect motor behaviour significantly differently. As previously observed for dyadic interactions (Worm et al. 2018b), it made no difference whether the dummy produced a static random playback sequence or a dynamic echo playback. The robot’s influence on the following behaviour and group dynamics was reliably observed with regard to nearest neighbour distances (Fig. 6) and the number of followed turns (Fig. 7) and declined with increasing group size. The strongest reactions were always observed in single fish, while larger groups hardly followed for extended time periods.

Entering an open, featureless, and unfamiliar environment constitutes potentially risky behaviour and is therefore avoided by many animals, including fish (Maximino et al. 2010). During control trials without the robot’s presence, *M. rume* clearly favoured the proximity of the tank walls (Fig. 5) and frequently grouped in the corners of the tank (Fig. 4). The latter behaviour may; however, reflect a tendency to seek shelter among conspecifics in an unfamiliar situation (Hamilton 1971) rather than a natural grouping habit of the test fish.

The declining attractiveness of the robot in larger groups that was observed during playback emission may reflect that the other live fish of the group were simply more attractive than the robot but could also hint at a quorum rule for decision-making in groups (Sumpter et al. 2008). Because the robot never moved as close to the tank walls as the fish, individuals had to weigh the social attraction towards the electrically signalling dummy against taking the risk to abandon the relative safety of the tank walls and the remaining group when following the dummy fish into the open area. Employing quorum rules can guard animal groups against bad decisions because the probability that misinformed decisions of a small number of individuals get amplified into a group response decreases with group size (Ward et al. 2008). This was demonstrated in sticklebacks (*Gasterosteus aculeatus*), who only took risky decisions as a collective if these were initiated by a certain proportion of the group, which meant that a second fish dummy was needed to induce collective swimming in larger groups (Ward et al. 2008).

We have previously reported results from a similar study with only single individuals encountering our mobile fish robot. In this study, interactive playback that simulated the echo latency of *M. rume* provoked more echo responses from live fish compared to static sequences of random IDIs. The discharge synchronizations the fish engaged in during these experiments were on average strongest when they followed after the dummy, maintained a parallel orientation, and reached a distance corresponding approximately to the outer limit of active electrolocation (Worm et al. 2018b).

In the current experiments involving small groups of fish in addition to the mobile dummy, we deployed a reversed approach to observe and analyse the exact behaviour that fish displayed during EOD synchronization of a predefined magnitude. During group interactions of two *M. rume* and the mobile fish robot, we frequently observed that fish responded to both electrical playback types of the dummy with discharge synchronizations. Such episodes of relatively strong discharge synchronization by *M. rume* frequently occurred in behavioural situations during which the individual that initiated synchronization approached either the robot or a conspecific as illustrated in Figs. 8 and 9. These approaches may signify attempts to communicate by the approaching fish and support our social attention hypothesis for electrical discharge synchronizations among mormyrids.

The mormyrid echo response has previously been considered to be either a jamming avoidance strategy during active electrolocation or a means of social communication (Heiligenberg 1976; Kramer 1974; Russel et al. 1974). According to our social attention hypothesis, social functions of echoing and jamming avoidance during active electrolocation are not mutually exclusive. On the neurophysiological level, afferent sensory input from knollenorgans is cancelled out by a corollary discharge signal in the hindbrain each time the fish produces an EOD (Bell and Grant 1989), which means that through the knollenorgan pathway only signals of other fish are detected and it can therefore be used for communication. Knollenorgans are, however, most likely also employed to locate and approach signalling conspecifics even when they are moving in complete darkness (Worm et al. 2018a; Hopkins 2005). Consequently, echoing of a conspecific's EOD may not only avoid jamming of the active sensory system but also avoid jamming of the passive electrosensory knollenorgans, and thus protect their function during spatial interactions.

Similar to the ‘electrosensory refractoriness avoidance’ described in the South American gymnotiform *Gymnotus* (Guariento et al. 2014), which is based on a strongly reduced sensitivity to electrical signals for a short period following each EOD in these functionally similar, but unrelated weakly electric fish (Westby 1975) echoing mormyrids ensure that a sender places its EOD after the period of reafferent inhibition of the receiver's knollenorgans, while simultaneously avoiding coincidence with the receiver’s next EOD. This refractoriness avoidance through echoing will therefore guarantee that the receiver can detect the signal generated by the sender. It also increases the likelihood that the receiver will notice that it is subject to social intentions by the fish that echoes its signals. In other words, echoing avoids jamming of the knollenorgan pathway of an approaching individual, while simultaneously assuring that the other individual realizes that it is being approached. Since this works in both directions, the approaching individual will notice it is being detected once the approached individual starts generating echo responses of its own, and thus engages in a mutual signal synchronization. This would result in a closed-loop electromotor 'action-response' communication (compared to Hurd and Enquist 2005), and thus enables individuals to mutually allocate social attention during electro-communication.

Two observations of our current study supported this social attention hypothesis. First, echoing occurred in bouts that resulted in brief episodes of relatively strong EOD synchronization and these episodes were frequently associated with approach behaviours (Fig. 10). In particular, *M. rume* would in these situations often approach either the robot or a conspecific by catching up from behind or converging to a lateral swimming position. On many occasions, the former behaviour transitioned into the latter, resulting in a stereotyped behavioural interaction pattern between two individuals. It is noteworthy in this context that the analysis of a sender’s EOD waveform is based on the comparison of signal inputs from knollenorgans distributed over the receiver’s body (Baker et al. 2013) and stereotypical approach behaviours may thus optimise the representation of communication signals. Furthermore, it has been experimentally demonstrated that mormyrids orient along the electrical field lines of another individual’s EOD during approach (Hopkins 2005) and that the presence of electric signals influences spatial interrelations during dyadic interactions (Worm et al. 2017, 2018a). The second observation that supports our hypothesis is that during episodes where synchronization resulted from mutual echoing among pairs of live fish, it was mostly the approaching individual that initiated this synchronization.

We therefore propose that the ability of mormyrids to selectively synchronize their signalling by mutually generating echo responses to each other's EODs (Arnegard and Carlson 2005; Gebhardt et al. 2012a), and the observation that the resulting episodes of synchronized discharge activity rapidly switch between specific individuals that interact within a group (Gebhardt et al. 2012b), represents a communication strategy that allows individual fish to address a particular individual to exchange information in a variety of social contexts. This would be achieved by a sender placing EODs into a sensitive window in the knollenorgan pathway of a designated receiver. This mechanism could establish a social link between mormyrids that depends on very fast and precise time-locking of the EODs of two individuals and enables electro-communication also in groups, where electrical noise is imposed on dyadic interactions by the signalling activity of conspecifics. This communication strategy would also have implications for the potential complexity of social dynamics among electro-communicating mormyrids.

*Mormyrus rume* belongs to a subgroup of mormyrids termed clade A, whose members have a neuroanatomical differentiation in the exterolateral nucleus of the midbrain that enables the processing of sub millisecond differences in the time course of the EOD waveforms of other individuals via the knollenorgan pathway (Carlson et al. 2011). Clade A mormyrids were shown to be capable of individual recognition based on EOD waveform (Hanika and Kramer 2005) and their EOD can also encode important identity information such as sex, reproductive state and dominance status (Terleph 2004; Carlson et al. 2000; Bass and Hopkins 1983). During encounters of two fish, the ability to synchronize electrical signalling by mutually responding to each other's EODs with echo responses may thus facilitate the evaluation of the physical condition and EOD waveform information of another individual, simultaneously. Jamming avoidance in the knollenorgan pathway could in these situations facilitate undisturbed mutual identification of individuals based on differences in EOD waveform, and facilitate the assessment of conspecifics through the exchange of dominance-related waveform information between unfamiliar individuals and thus determine hierarchy ranks without unnecessarily costly conflicts (Parker 1974; Enquist and Leimar 1983).

The animals in our study were phenotypically juvenile and of undetermined gender. However, sex-related waveform differences have also been described for the genus *Mormyrus* (Kramer 2013) and mutual discharge synchronizations could thus facilitate mate choice based on waveform information in mature individuals. In some species, especially male EOD duration may serve as a fitness indicator, since the increased waveform duration in mature males can be interpreted as a handicap signal (Zahavi 1975) that leaves them more vulnerable to predation by electrosensory catfish, who can detect the higher amount of low-frequency components contained in long-duration EODs (Hanika and Kramer 2000). Indeed, extensive EOD regularisations have for instance been observed during courtship of the mormyrid *Marcusenius macrolepidotus,* although the temporal interrelations of their respective signalling sequences were not analysed (Werneyer and Kramer 2005). Further studies could thus investigate differential responses based on identity information encoded into the playback EOD or the identity of the receiving animals in closed-loop behavioural experiments.

While most mormyrids are invertivores that mostly prey on insect larvae (Kouamélan et al. 1999), and many clade A species show territorial and aggressive behaviours, particularly in the breeding season (Carlson 2016; Friedman and Hopkins 1996), field reports from predatory *Mormyrops anguilloides* have shown that these mormyrids gather in relatively stable groups and hunt in packs for small cichlids (Arnegard and Carlson 2005). Based on their observations of cooperative hunting groups in Lake Malawi, (Arnegard and Carlson 2005) hypothesized that mutual synchronization of bursts through echoing allows 'mutual acknowledgement of recognition' between individuals of the group. Generating social attention via electric signal synchronization could thus facilitate a wide range of social behaviours from aggressive territorial displays and mate choice to shoaling and even cooperative hunting in mormyrids.

The acceptance of robotic group members may not only depend on certain aspects of their chemical, visual or behavioural appearance. In addition, animals may expect or anticipate a response to their own action (Landgraf et al. 2016). If the interaction partner (i.e. the robot) does not react appropriately, the animal perceives a discrepancy between expectation and actual observation. In effect, the robot’s acceptance by the group, or the effect of a focal behaviour may be diminished.

The fact that the echo response did not yield measurable differences in the movement patterns of live fish compared to the random EOD setting may therefore be explained by the inability of the robot to produce echo responses with respect to the particular temporal and spatial contexts. It is conceivable that the synchronization episodes convey specific meanings that depend on who initiates them, from which relative position, for how long and so on. Secondly, specific locomotor behaviours throughout or after the synchronization episode may affect the interaction partner only if the robotic behaviour matches a certain expected pattern. The echo response in this study was implemented as a hard-wired circuit and locomotory control was exercised manually in both treatments. Closing the feedback loop and making the robot fully interactive also on a locomotor level will allow us to generate and investigate more complex behavioural responses experimentally. This will eventually help us to further elucidate the social implications of the mormyrid echo response by focussing on the behaviours occurring during and immediately after electrical synchronizations events depending on e.g. familiarities, gender, and relative dominance relationships as communicated by the waveform of the EOD.
